# Crystal structure of a pyrazine-2,3-dicarboxamide ligand and of its silver(I) nitrate complex, a three-dimensional coordination polymer

**DOI:** 10.1107/S2056989017006387

**Published:** 2017-05-05

**Authors:** Dilovan S. Cati, Helen Stoeckli-Evans

**Affiliations:** a Debiopharm International S.A., Chemin Messidor 5-7, CP 5911, CH-1002 Lausanne, Switzerland; b Institute of Physics, University of Neuchâtel, rue Emile-Argand 11, CH-2000 Neuchâtel, Switzerland

**Keywords:** crystal structure, carboxamide, pyrazine, pyridine, silver(I), Ag—Ag bond, three-dimensional coordination polymer, hydrogen bonding

## Abstract

The title ligand, C_18_H_16_N_6_O_2_·2H_2_O (**L1**) [*N* ^2^,*N* ^3^-bis­(pyridin-4-ylmeth­yl)pyrazine-2,3-dicarboxamide], crystallized as a dihydrate. The mol­ecule is U-shaped with the carboxamide groups being *cis* to one another, making a dihedral angle of 81.6 (5)°. The terminal pyridine rings are inclined to one another by 58.5 (4)°. There is an intra­molecular N—H⋯N_pyrazine_ hydrogen bond present, forming an *S*(5) ring motif. In the crystal, adjacent mol­ecules are linked by N—H⋯O_carboxamide_ hydrogen bonds, forming a chain along [001]. A chain of hydrogen-bonded water mol­ecules is linked to the chain of (**L1**) mol­ecules by O—H⋯N hydrogen bonds, forming columns propagating along the *c* axis. The columns are linked by C—H⋯O and C—H⋯N hydrogen bonds, forming a three-dimensional supra­molecular structure. The reaction of ligand (**L1**) with silver(I) nitrate led to the formation of a new three-dimensional coordination polymer, {[Ag(C_18_H_16_N_6_O_2_)]NO_3_}_*n*_, poly[[[μ_4_-*N* ^2^,*N* ^3^-bis­(pyridin-4-ylmeth­yl)pyrazine-2,3-dicarboxamide]­silver(I)] nitrate] (**I**). The asymmetric unit is composed of half of one silver ion, located on a twofold rotation axis, half a ligand mol­ecule and half a positionally disordered nitrate anion located about a twofold rotation axis. The full mol­ecule of the ligand is generated by twofold rotational symmetry, with this twofold axis bis­ecting the C_ar_—C_ar_ bonds of the pyrazine ring and the Ag—Ag bond. The carboxamide groups are now *trans* to one another, making a dihedral angle of 65.8 (4)°. The two terminal pyridine rings are inclined to one another by 6.6 (3)°. Two ligands wrap around an Ag—Ag bond of 3.1638 (11) Å, forming a figure-of-eight-shaped complex mol­ecule. Each silver ion is coordinated by two pyridine N atoms and by two carboxamide O atoms of neighbouring mol­ecules, hence forming a three-dimensional framework. The nitrate anion is linked to the framework by N—H⋯O and C—H⋯O hydrogen bonds.

## Abstract 2

The reaction of the ligand *N* ^2^,*N* ^3^-bis­(pyridin-4-ylmeth­yl)pyrazine-2,3-dicarboxamide with silver(I) nitrate led to the formation of a three-dimensional coordination polymer.

## Chemical context   

The title ligand, *N* ^2^,*N* ^3^-bis­(pyridin-4-ylmeth­yl)pyrazine-2,3-dicarboxamide (**L1**), is one of a series of ligands synthesized in order to study the superexchange in supra­molecular complexes formed using pyrazine carboxamide derivatives and first row transition metal ions (Cati, 2002 ▸; Cati *et al.*, 2004 ▸). To the best of our knowledge, neither the synthesis nor the crystal structure of (**L1**) have been described previously. It is very similar to the ligand *N* ^2^,*N* ^3^-bis­(pyridin-2-ylmeth­yl)pyrazine-2,3-dicarboxamide (**L2**), for which a number of transition metal complexes have been described, including some inter­esting tetra­nuclear 2×2 grid-like and square complexes (Hausmann *et al.*, 2003 ▸; Klingele *et al.*, 2007 ▸). Two such complexes, {[Cu_4_(**L2**)_4_](ClO_4_)_4_}·5CH_3_OH·4H_2_O and {[Ni_4_(**L2**)_4_]Cl_4_}·5CH_3_CN·13H_2_O (Cati *et al.*, 2004 ▸), exhibit anion encapsulation, and magnetic susceptibility measurements indicate that they are weakly anti-ferromagnetic, with *J* values of −5.87 and −2.64 cm^−1^, respectively.

A search of the Cambridge Structural Database (CSD; Groom *et al.*, 2016 ▸), indicated that silver nitrate is an excellent metal salt for the formation of multi-dimensional coordination polymers. The silver ion can have multiple coordination geometries and modes, and the nitrate anion has been shown to coordinate to metal ions in a number of different modes, many of which involve bridging metal ions. The properties of the complexes formed are extremely varied. For example, with the tetra­dendate ligand 1,6-bis­(2*H*-1,2,3-triazol-2-yl)hexane, Huo *et al.* (2016 ▸) synthesized the three-dimensional coordin­ation polymer, *catena*-[[μ-2,2′-(butane-1,4-di­yl)bis­(2*H*-1,2,3-triazole)]bis­(μ-nitrato)disilver]. They showed that it exhibits highly selective and sensitive luminescence sensing of Cr_2_O_7_^2−^ ions in aqueous solution. With the rigid tripodal arene-core-based nitro­gen ligand, 1,3,5-tris­(pyrazol-1-yl)benzene, Shu *et al.* (2006 ▸) formed a porous metal–organic framework, *viz. catena*-[bis­(μ_3_-nitrato-*O*,*O*′,*O*′′)bis­(μ_3_-1,3,5-tris­(pyrazol-1-yl)benzene-*N*,*N*′,*N*′′)tris­ilver(I) nitrate]. The nitrate counter-anions located in the cationic framework can be exchanged reversibly without destruction of the structure. Hence, this compound can act as a zeolite-like porous material for anion exchange.
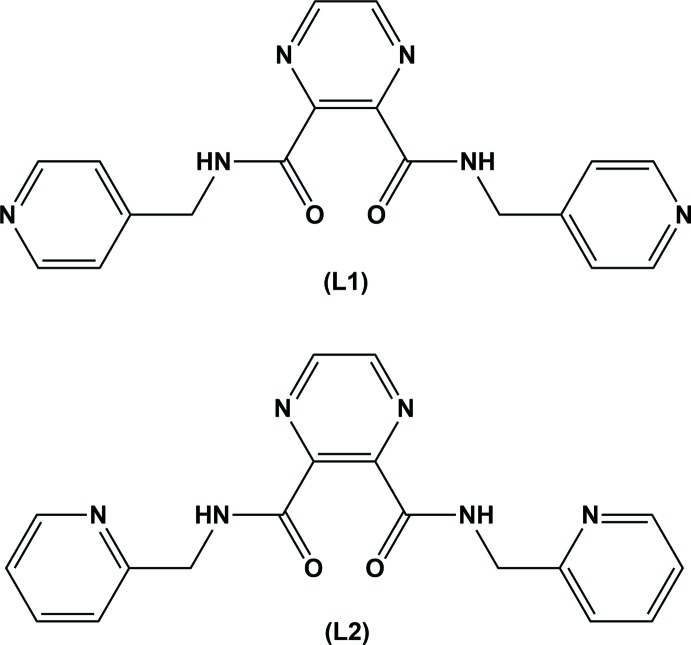


The title ligand has potentially two bidentate (N,N) and two monodentate (N_pyridine_) coordination sites. It is therefore an inter­esting ligand to study its coordination behaviour with silver nitrate, and herein, we describe the solid state structures of ligand (**L1**), and the new three-dimensional coordination polymer, poly[[[μ_4_-*N* ^2^,*N* ^3^-bis­(pyridin-4-ylmeth­yl)pyrazine-2,3-dicarboxamide]­silver(I)]nitrate] (**I**).

## Structural commentary   

**Figure 1:**
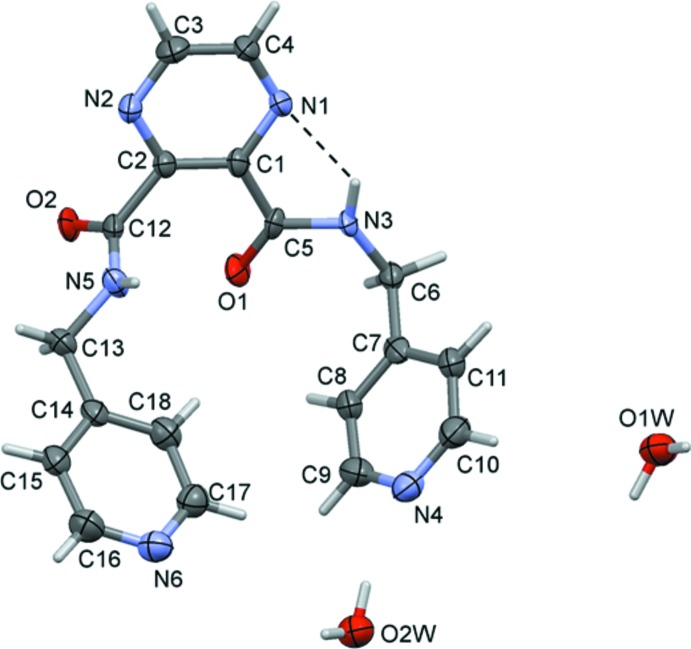
A view of the mol­ecular structure of ligand (**L1**), with atom labelling. Displacement ellipsoids are drawn at the 50% probability level. The intra­molecular N—H⋯N contact is shown as a dashed line (see Table 2 ▸).

The title ligand (**L1**) crystallized as a dihydrate, and its mol­ecular structure is illustrated in Fig. 1 ▸. The mol­ecule is U-shaped with the carboxamide groups (C6/N3/C5/O1) being *cis* to one another, making a dihedral angle of 81.6 (5)°. The terminal pyridine rings (N4/C7–C11) are inclined to one another by 58.5 (4)°. There is an intra­molecular N—H⋯N hydrogen bond present, forming an *S*(5) ring motif (Fig. 1 ▸ and Table 1 ▸).
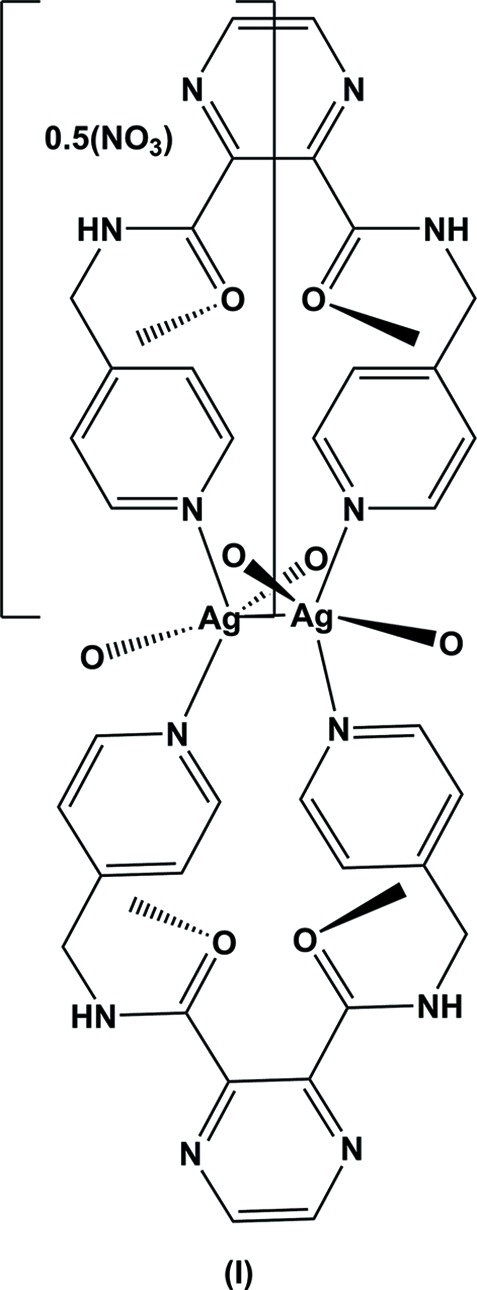


**Figure 2:**
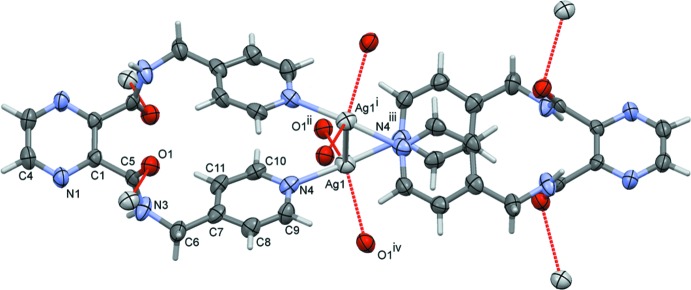
A view of the figure-eight arrangement of the title complex (**I**), with atom labelling for the asymmetric unit and some symmetry-related atoms (see Table 1 ▸ for details). The unlabelled atoms of the ligand on the left-hand-side of the figure are related to the labelled atoms by twofold rotational symmetry (symmetry operation: −*x* + 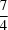, −*y* + 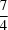, *z*). The nitrate anions have been omitted for clarity.

**Figure 3:**
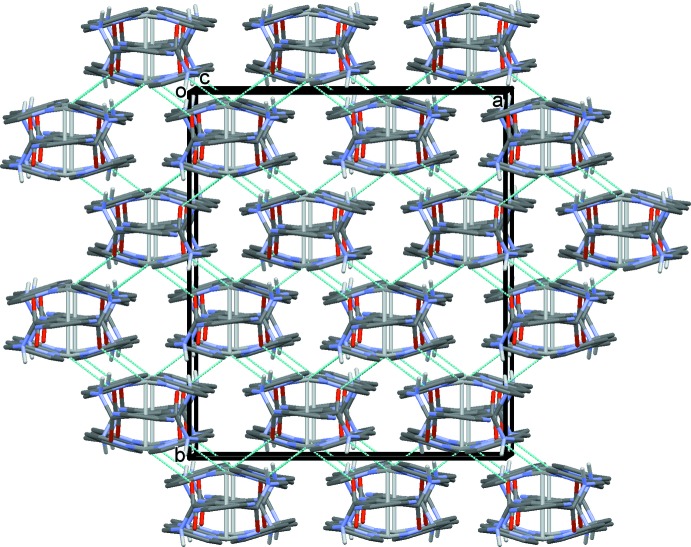
A view along the *c* axis of the three-dimensional framework of complex (**I**), showing the Ag⋯O bonds as dashed lines (see Table 2 ▸). The nitrate anions and the C-bound H atoms have been omitted for clarity.

The reaction of the ligand with silver(I) nitrate led to the formation of a three-dimensional coordination polymer (**I**). The coordination of the ligand to the silver ions is illustrated in Fig. 2 ▸. Selected bond lengths and angles in (**I**) are given in Table 2 ▸. The asymmetric unit is composed of a silver ion, located on a twofold rotation axis, half a ligand mol­ecule and half a nitrate anion. The full mol­ecule of the ligand is generated by twofold rotational symmetry, with this twofold axis bis­ecting the C4—C4^i^ bonds of the pyrazine ring and the Ag1—Ag1^i^ bond (Table 2 ▸). The carboxamide groups (C6/N3/C5/O1) are now *trans* to one another, making a dihedral angle of 65.8 (4)°. The terminal pyridine rings (N4/C7–C11) are inclined to one another by 6.6 (3)°. Two ligands effectively wrap around a Ag—Ag bond of 3.1638 (11) Å, forming a figure-of-eight-shaped mol­ecule, with each silver ion being coordinated by two pyridine N atoms. The silver ions are each further coordinated by the carboxamide O atom, O1, of neighbouring mol­ecules, hence forming a three-dimensional framework, illustrated in Fig. 3 ▸. If one considers that the silver ion, Ag1, is fivefold coordinate (N_2_O_2_Ag^i^) then its coordination sphere can be described as distorted trigonal–bipyramidal, with a τ_5_ value of 0.8 (τ_5_ = 1 for perfect trigonal–pyramidal geometry and 0 for perfect square-pyramidal geometry; Addison *et al.*, 1984 ▸). However, if one considers the Ag1 ion to be fourfold coordinate, N_2_O_2_, with a τ_4_ value of 0.55, its coordination sphere can be described as inter­mediate between trigonal–pyramidal and seesaw (τ_4_ = 1 for a perfect tetra­hedral geometry and 0 for a perfect square-planar geometry. For inter­mediate structures, including trigonal–pyramidal and seesaw, τ_4_ falls within the range of 0 to 1; Yang *et al.*, 2007 ▸). The nitrate anion that does not coordinate to the silver(I) ion is positionally disordered, and also located about a twofold rotation axis.

## Supra­molecular features   

**Figure 4:**
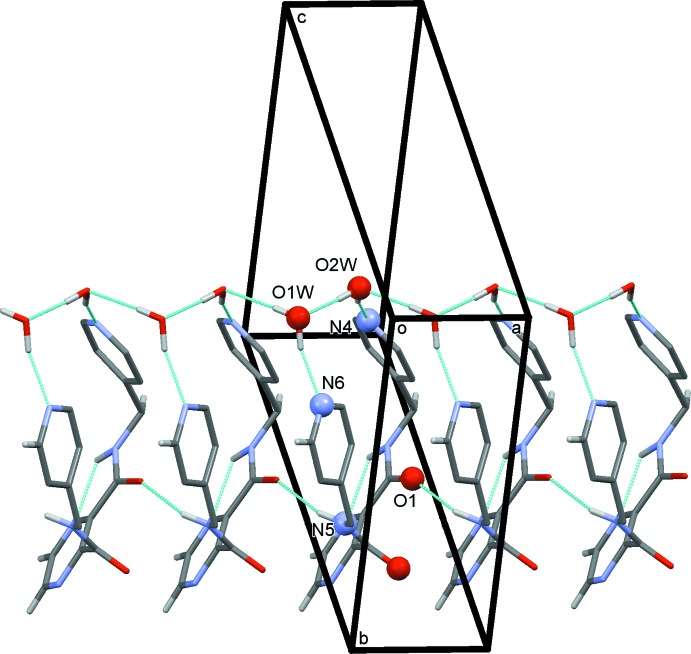
A partial view along direction [111] of the crystal packing of ligand (**L1**). The hydrogen bonds are shown as dashed lines (see Table 1 ▸)

**Figure 5:**
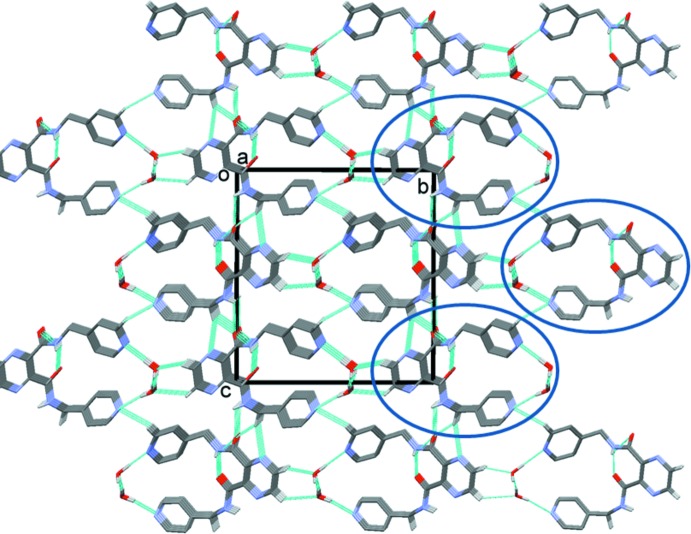
A view along the *a* axis of the crystal packing of ligand (**L1**). The columns of (**L1**) mol­ecules, linked by hydrogen bonds involving the water mol­ecules, are indicated by blue circles. The hydrogen bonds are shown as dashed lines (see Table 1 ▸), and for clarity, only the H atoms involved in hydrogen bonding have been included.

In the crystal of ligand (**L1**), mol­ecules are linked by N—H⋯O(water) hydrogen bonds forming chains propagating along the *c-*axis direction (Table 1 ▸ and Fig. 4 ▸). Parallel to this chain of mol­ecules is a chain of hydrogen-bonded water mol­ecules (Table 1 ▸ and Fig. 4 ▸), which is linked to the chain of (**L1**) mol­ecules by O—H⋯N hydrogen bonds, forming columns propagating along the *c* axis. The columns are linked by C—H⋯O and C—H⋯N hydrogen bonds, forming a three-dimensional supra­molecular structure (Table 1 ▸ and Fig. 5 ▸).

**Figure 6:**
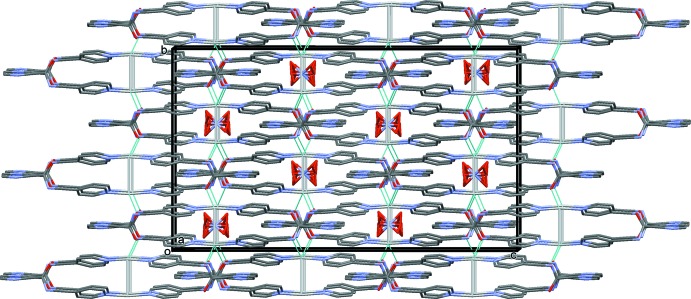
A view along the *a* axis of the crystal packing of complex (**I**), showing the Ag⋯O bonds as dashed lines (see Table 2 ▸). For clarity, all H atoms have been omitted.

In (**I**), the nitrate anion is situated in the cavities of the three-dimensional framework and is linked to the framework by N—H⋯O and C—H⋯O hydrogen bonds (Table 3 ▸ and Fig. 6 ▸). The nitrate anion in (**I**) is not essential for forming the three-dimensional structure, although it may act as a template for the formation of the framework (Batten *et al.*, 2009 ▸). This is in contrast to the MOF *catena*-[bis­(μ_3_-nitrato-*O,O′,O′′*)bis(μ_3_-1,3,5-tris­(pyrazol-1-yl)benzene-*N,N′,N′′*)tris­ilver(I) nitrate] mentioned above (Shu *et al.*, 2006 ▸), in which there are nitrate anions coordinating the silver ions in a μ_3_ fashion and present also in the framework cavities. There are, of course, other examples reported in the Cambridge Structural Database (Groom *et al.*, 2016 ▸).

In describing compound (**I**) as a three-dimensional coordination polymer, we make here the distinction between a coordination polymer and a metal–organic framework. Both have a three-dimensional framework but there are no cavities, even small ones, in the structure of (**I**). Hence, it should be classed as a three-dimensional coordination polymer according to the IUPAC recommendations on the ‘Terminology of metal–organic frameworks and coordination polymers’ (Batten *et al.*, 2013 ▸).

## Database survey   

A search of the Cambridge Structural Database (Version 5.38, update February 2017; Groom *et al.*, 2016 ▸) for Ag–Ag complexes, excluding silver ion clusters of any kind, gave 321 hits. Limiting the search to Ag–Ag complexes with each silver ion coordinated by two pyridine N atoms, gave 95 hits. The Ag—Ag distances vary between *ca* 2.6–3.6 Å. One compound, bis­[μ_2_-2,7-di-*tert*-butyl-9,9-dimethyl-*N*,*N*′-bis­[(3-pyrid­yl)meth­yl]xanthene-4,5-dicarboxamide]­disilver bis­(tri­fluoro­methane­sulfonate) chloro­form solvate (HIFKUD; Yue *et al.*, 2007 ▸), is particularly inter­esting because it too involves a dicarboxamide ligand, *viz. N*,*N*′-bis­[(3-pyrid­yl)methy]xanth­ene-4,5-dicarboxamide), that wraps around an Ag—Ag bond forming a similar figure-of-eight-shaped complex. Here the Ag—Ag bond length is 3.134 (1) Å, slightly shorter than the value of 3.1638 (11) Å observed in (**I**); Table 2 ▸. A search for the benzene analogue of ligand (**L1**), *N*-(4-pyridyl­meth­yl)carbamo­yl)benzene, gave only two hits. Both of them are mercury(II) complexes, *viz.* the binuclear complex bis­{μ_2_-1,2-bis­[*N*-(4-pyridyl­meth­yl)carbamo­yl]benzene}­tetra­kis­(tri­fluoro­acetato)­dimercury(II) methanol solvate (XAHSIJ; Burchell *et al.*, 2004 ▸) and the two-dimensional network *catena*-[bis­{μ_2_-1,2-bis­[*N*-(4-pyridyl­meth­yl)carbamo­yl]benzene}­dichlorido­mercury(II) 1,2-di­chloro­ethane solvate] (XAHSOP; Burchell *et al.*, 2004 ▸). A search for the benzene analogue of ligand (**L2**), [*N*-(2-pyridyl­meth­yl)carbamo­yl]benzene, gave zero hits, while that for [*N*-(3-pyridyl­meth­yl)carbamo­yl]benzene gave eight hits. The latter includes the crystal structure of the dihydrate of the ligand itself (PANROM; Ge *et al.*, 2005 ▸) and the structures of seven first-row transition metal one-, two- and three-dimensional coordination polymers.

## Synthesis and crystallization   

Ligand (**L1**) was prepared using the same procedure as for ligand (**L2**) (Cati *et al.*, 2004 ▸). Dimethyl pyrazine-2,3-di­carboxyl­ate (1.96 g, 10 mmol; Alvarez-Ibarra *et al.*, 1994 ▸) and an excess of 4-(amino­meth­yl)pyridine (3.24 g, 30 mmol) in 35 ml of methanol were heated to reflux and heating was continued for 72 h in a two-necked flask (100 ml). The brown solution that formed was concentrated and 15 ml of water were added, which precipitated qu­anti­tatively ligand (**L1**). The solid was collected by filtration, washed with 10 ml of water and dried in air. Recrystallization in ethanol gave colourless plate-like crystals (yield is qu­anti­tative; m.p. 474 K). Spectroscopic data: ^1^H NMR (400 MHz, DMSO-*d*6): 9.33 (*t*, 1H, *J*_hg_ = 6.1, Hh); 8.86 (*s*, 1H, H*n* = H*m*); 8.49 (*dd*, 2H, *J*_ba_ = 4.5, *J*_be_ = 1.5, H*b* = H*d*); 7.39 (*dd*, 2H, *J*_ab_ = 4.5, *J*_eb_ = 1.5, H*a* = H*e*); 4.52 (*d*, 2H, *J*_gh_ = 6.1, Hg). ^13^C NMR (400 MHz, DMSO-*d*6): 165.8, 150.3, 148.9, 147.6, 145.6, 123.0, 42.2. IR (KBr pellet, cm^−1^): 3273 (*s*), 3031 (*s*), 1675 (*vs*), 1602 (*vs*), 1564 (*vs*), 1520 (*vs*), 1416 (*vs*), 1364 (*s*), 1311 (*s*), 1292 (*s*), 1220 (*s*), 1185 (*m*), 1164 (*m*), 1124 (*s*), 1069 (*m*), 995 (*s*), 871 (*w*), 830 (*m*), 787 (*m*), 745 (*m*), 715 (*m*), 611 (*m*), 575 (*w*), 504 (*m*), 495 (*m*), 475 (*m*). Elemental analysis for [C_18_H_16_N_6_O_2_]·H_2_O (*M*_r_ = 366.39 g mol^−1^): calculated: C: 59.01 H: 4.95 N: 22.94%; found: C: 59.10 H: 5.05 N: 23.10%.

Complex (**I**): A solution of (**L1**) (46 mg; 0.126 mmol) in 6 ml CHCl_3_ was introduced into a 13 mm diameter glass tube. It was layered with methanol (*ca* 2 ml) used as a buffer zone. A solution of AgNO_3_ (21 mg, 0.126 mmol) in MeOH (6 ml) was then added gently to avoid possible mixing. The glass tube was sealed with a perforated parafilm and left at room temperature. Colourless block-like crystals were obtained after a few days (yield 60 mg, 92%). Elemental analysis for AgC_18_H_16_N_7_O_5_: (*M*_r_ = 518.25 g mol^−1^): calculated: C: 41.72 H: 3.11 N: 18.92%; found: C: 41.65 H: 3.09 N: 18.85%.

## Refinement   

Crystal data, data collection and structure refinement details are summarized in Table 4 ▸. For the ligand (**L1**), the NH and water H atoms were located in difference-Fourier maps and refined with distance restraints: O—H = 0.85 (2) Å, N—H = 0.88 (2) Å with *U*_iso_(H) = 1.5*U*_eq_(O) and 1.2*U*_eq_(N). In the final cycles of refinement, the water H atoms were treated as riding atoms. For complex (**I**), the NH H atoms were included in calculated positions and treated as riding: N—H = 0.88 Å with *U*_iso_(H) = 1.2*U*_eq_(N). For both compounds, the C-bound H atoms were included in calculated positions and refined as riding: C—H = 0.95–0.99 Å with *U*_iso_(H) = 1.2*U*_eq_(C). The nitrate anion is positionally disordered about a twofold rotation axis and was refined with fixed occupancies (N10*A* and N10*B* = 0.5, O11 and O13 = 0.5, O12 and O14 = 0.25), and all their ADP’s were made equal to that of atom O11. Using a one-circle image-plate diffraction system it is not possible to measure 100% of the Ewald sphere, particularly for triclinic or monoclinic systems. This is the case for ligand (**L1**), which crystallized in the monoclinic space group *Pc* and for which only 94.7% of the Ewald sphere was accessible.

## Supplementary Material

Crystal structure: contains datablock(s) L1, I, Global. DOI: 10.1107/S2056989017006387/pj2044sup1.cif

Structure factors: contains datablock(s) L1. DOI: 10.1107/S2056989017006387/pj2044L1sup2.hkl

Structure factors: contains datablock(s) I. DOI: 10.1107/S2056989017006387/pj2044Isup3.hkl

Supporting information file. DOI: 10.1107/S2056989017006387/pj2044L1sup4.cml

CCDC references: 1546645, 1546644

Additional supporting information:  crystallographic information; 3D view; checkCIF report

## Figures and Tables

**Table 1:** Hydrogen-bond geometry (Å, °) for (**L1**)

*D*—H⋯*A*	*D*—H	H⋯*A*	*D*⋯*A*	*D*—H⋯*A*
N3—H3*N*⋯N1	0.88 (3)	2.08 (5)	2.718 (7)	129 (5)
N5—H5*N*⋯O1^i^	0.88 (3)	2.05 (4)	2.858 (7)	151 (6)
O1*W*—H1*WA*⋯O2*W* ^ii^	0.86	1.91	2.762 (7)	177
O1*W*—H1*WB*⋯N6^iii^	0.94	1.97	2.886 (7)	164
O2*W*—H2*WA*⋯O1*W* ^iv^	0.86	1.91	2.765 (8)	172
O2*W*—H2*WB*⋯N4	0.85	2.06	2.888 (7)	164
C3—H3⋯O1*W* ^v^	0.95	2.38	3.273 (8)	156
C4—H4⋯O2*W* ^vi^	0.95	2.58	3.253 (8)	128
C6—H6*A*⋯N2^vii^	0.99	2.57	3.515 (8)	159
C16—H16⋯N4^iv^	0.95	2.60	3.451 (9)	149

Symmetry codes: (i) 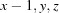; (ii) 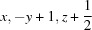; (iii) 
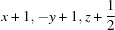
; (iv) 
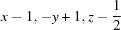
; (v) 
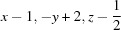
; (vi) 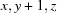; (vii) 
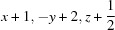
.

**Table 2:** Selected geometric parameters (Å, °) for (**I**)

Ag1—Ag1^i^	3.1638 (11)	Ag1—O1^ii^	2.814 (5)
Ag1—N4	2.109 (5)		
			
N4—Ag1—N4^iii^	173.0 (2)	O1^ii^—Ag1—N4	97.80 (15)
O1^ii^—Ag1—O1^iv^	109.48 (14)	O1^iv^—Ag1—N4	86.26 (15)
Ag1^i^—Ag1—N4	86.51 (14)	Ag1^i^—Ag1—O1^ii^	125.26 (10)

Symmetry codes: (i) 
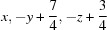
; (ii) 
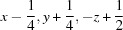
; (iii) 
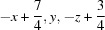
; (iv) 
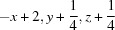
.

**Table 3:** Hydrogen-bond geometry (Å, °) for (**I**)

*D*—H⋯*A*	*D*—H	H⋯*A*	*D*⋯*A*	*D*—H⋯*A*
N3—H3*N*⋯O11^v^	0.88	1.86	2.744 (14)	178
N3—H3*N*⋯O13^v^	0.88	2.26	2.875 (13)	127
C4—H4⋯O11^vi^	0.95	2.45	3.378 (13)	165
C4—H4⋯O14^vii^	0.95	2.40	3.33 (2)	168
C9—H9⋯O13^viii^	0.95	2.50	3.224 (14)	133
C11—H11⋯O13^v^	0.95	2.51	3.154 (13)	126

Symmetry codes: (v) 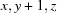; (vi) 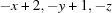; (vii) 
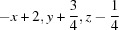
; (viii) 
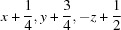
.

**Table 4:** Experimental details

	(**L1**)	(**I**)
Crystal data		
Chemical formula	C_18_H_16_N_6_O_2_·2H_2_O	[Ag(C_18_H_16_N_6_O_2_)]NO_3_
*M*_r_	384.40	518.25
Crystal system, space group	Monoclinic, *P* *c*	Orthorhombic, *F* *d* *d* *d*
Temperature (K)	153	153
*a*, *b*, *c* (Å)	4.3677 (6), 14.0232 (12), 15.1816 (18)	14.9776 (16), 17.3228 (12), 29.570 (4)
α, β, γ (°)	90, 96.153 (16), 90	90, 90, 90
*V* (Å^3^)	924.50 (19)	7672.1 (14)
*Z*	2	16
Radiation type	Mo *K*α	Mo *K*α
μ (mm^−1^)	0.10	1.10
Crystal size (mm)	0.50 × 0.15 × 0.05	0.40 × 0.30 × 0.20

Data collection		
Diffractometer	Stoe IPDS 1	Stoe IPDS 1
Absorption correction	Multi-scan (*MULABS*; Spek, 2009 ▸)	Multi-scan (*MULABS*; Spek, 2009 ▸)
*T*_min_, *T*_max_	0.763, 1.000	0.985, 1.000
No. of measured, independent and observed [*I* > 2σ(*I*)] reflections	7134, 3388, 1693	13548, 1721, 1038
*R*_int_	0.107	0.096
(sin θ/λ)_max_ (Å^−1^)	0.615	0.600

Refinement		
*R*[*F* ^2^ > 2σ(*F* ^2^)], *wR*(*F* ^2^), *S*	0.051, 0.122, 0.77	0.045, 0.107, 0.90
No. of reflections	3388	1721
No. of parameters	260	141
No. of restraints	8	0
H-atom treatment	H atoms treated by a mixture of independent and constrained refinement	H-atom parameters constrained
Δρ_max_, Δρ_min_ (e Å^−3^)	0.21, −0.25	0.68, −0.65

Computer programs: *EXPOSE*, *CELL* and *INTEGRATE* in *IPDS-I* (Stoe & Cie, 2004 ▸), *SHELXS2014* (Sheldrick, 2008 ▸), *Mercury* (Macrae *et al.*, 2008 ▸), *SHELXL2014* (Sheldrick, 2015 ▸), *PLATON* (Spek, 2009 ▸) and *publCIF* (Westrip, 2010 ▸).

## supplementary crystallographic information

### (L1) N2,N3-Bis(pyridin-4-ylmethyl)pyrazine-2,3-dicarboxamide Crystal data

**Table:**

C_18_H_16_N_6_O_2_·2H_2_O	*F*(000) = 404
*M**_r_* = 384.40	*D*_x_ = 1.381 Mg m^−^^3^
Monoclinic, *P**c*	Mo *K*α radiation, λ = 0.71073 Å
*a* = 4.3677 (6) Å	Cell parameters from 3264 reflections
*b* = 14.0232 (12) Å	θ = 2.0–25.9°
*c* = 15.1816 (18) Å	µ = 0.10 mm^−^^1^
β = 96.153 (16)°	*T* = 153 K
*V* = 924.50 (19) Å^3^	Plate, colourless
*Z* = 2	0.50 × 0.15 × 0.05 mm

### (L1) N2,N3-Bis(pyridin-4-ylmethyl)pyrazine-2,3-dicarboxamide Data collection

**Table:**

Stoe IPDS 1 diffractometer	3388 independent reflections
Radiation source: fine-focus sealed tube	1693 reflections with *I* > 2σ(*I*)
Plane graphite monochromator	*R*_int_ = 0.107
φ rotation scans	θ_max_ = 25.9°, θ_min_ = 2.0°
Absorption correction: multi-scan (MULABS; Spek, 2009)	*h* = −5→5
*T*_min_ = 0.763, *T*_max_ = 1.000	*k* = −17→16
7134 measured reflections	*l* = −18→18

### (L1) N2,N3-Bis(pyridin-4-ylmethyl)pyrazine-2,3-dicarboxamide Refinement

**Table:**

Refinement on *F*^2^	Secondary atom site location: difference Fourier map
Least-squares matrix: full	Hydrogen site location: mixed
*R*[*F*^2^ > 2σ(*F*^2^)] = 0.051	H atoms treated by a mixture of independent and constrained refinement
*wR*(*F*^2^) = 0.122	*w* = 1/[σ^2^(*F*_o_^2^) + (0.0453*P*)^2^] where *P* = (*F*_o_^2^ + 2*F*_c_^2^)/3
*S* = 0.77	(Δ/σ)_max_ < 0.001
3388 reflections	Δρ_max_ = 0.21 e Å^−^^3^
260 parameters	Δρ_min_ = −0.25 e Å^−^^3^
8 restraints	Extinction correction: (SHELXL2016; Sheldrick, 2015), Fc^*^=kFc[1+0.001xFc^2^λ^3^/sin(2θ)]^-1/4^
Primary atom site location: structure-invariant direct methods	Extinction coefficient: 0.036 (6)

### (L1) N2,N3-Bis(pyridin-4-ylmethyl)pyrazine-2,3-dicarboxamide Special details

**Table:**

Geometry. All esds (except the esd in the dihedral angle between two l.s. planes) are estimated using the full covariance matrix. The cell esds are taken into account individually in the estimation of esds in distances, angles and torsion angles; correlations between esds in cell parameters are only used when they are defined by crystal symmetry. An approximate (isotropic) treatment of cell esds is used for estimating esds involving l.s. planes.

### (L1) N2,N3-Bis(pyridin-4-ylmethyl)pyrazine-2,3-dicarboxamide Fractional atomic coordinates and isotropic or equivalent isotropic displacement parameters (Å^2^)

**Table:**

	*x*	*y*	*z*	*U*_iso_*/*U*_eq_	
O1	0.7750 (11)	0.9108 (3)	0.4491 (3)	0.0358 (13)	
O2	0.5478 (11)	0.9994 (3)	0.2578 (3)	0.0351 (12)	
N1	0.4263 (14)	1.1135 (3)	0.5363 (4)	0.0321 (15)	
N2	0.1716 (14)	1.1331 (4)	0.3588 (4)	0.0330 (15)	
N3	0.7147 (13)	0.9491 (3)	0.5921 (3)	0.0258 (14)	
H3N	0.612 (13)	0.998 (3)	0.609 (4)	0.031*	
N4	0.4923 (14)	0.5966 (4)	0.6225 (4)	0.0403 (16)	
N5	0.1699 (14)	0.9119 (4)	0.3107 (4)	0.0313 (14)	
H5N	0.002 (11)	0.906 (5)	0.337 (4)	0.038*	
N6	−0.0999 (16)	0.5615 (4)	0.3180 (4)	0.0436 (17)	
C1	0.4668 (15)	1.0457 (4)	0.4743 (4)	0.0267 (17)	
C2	0.3401 (16)	1.0564 (4)	0.3878 (4)	0.0258 (16)	
C3	0.1396 (19)	1.1999 (5)	0.4209 (5)	0.043 (2)	
H3	0.030625	1.256737	0.403978	0.051*	
C4	0.2586 (18)	1.1888 (4)	0.5079 (5)	0.0360 (19)	
H4	0.219526	1.236935	0.549295	0.043*	
C5	0.6613 (15)	0.9624 (4)	0.5039 (4)	0.0280 (16)	
C6	0.9192 (16)	0.8748 (4)	0.6312 (5)	0.0290 (17)	
H6A	0.985533	0.891453	0.693707	0.035*	
H6B	1.105193	0.872221	0.599314	0.035*	
C7	0.7688 (16)	0.7778 (4)	0.6278 (5)	0.0307 (17)	
C8	0.8016 (18)	0.7151 (4)	0.5602 (5)	0.0376 (19)	
H8	0.917205	0.733033	0.513344	0.045*	
C9	0.669 (2)	0.6270 (5)	0.5600 (5)	0.046 (2)	
H9	0.702062	0.584555	0.513239	0.056*	
C10	0.4698 (19)	0.6573 (5)	0.6889 (5)	0.044 (2)	
H10	0.357252	0.636975	0.735668	0.053*	
C11	0.5967 (17)	0.7473 (5)	0.6950 (5)	0.0374 (19)	
H11	0.568042	0.787610	0.743743	0.045*	
C12	0.3639 (17)	0.9848 (4)	0.3138 (4)	0.0297 (17)	
C13	0.1367 (17)	0.8444 (4)	0.2372 (5)	0.0337 (18)	
H13A	0.332934	0.841179	0.210178	0.040*	
H13B	−0.024426	0.867753	0.191430	0.040*	
C14	0.0529 (17)	0.7469 (4)	0.2655 (4)	0.0303 (17)	
C15	−0.1501 (17)	0.6908 (5)	0.2141 (5)	0.0362 (18)	
H15	−0.242090	0.714271	0.158778	0.043*	
C16	−0.224 (2)	0.6001 (5)	0.2413 (5)	0.049 (2)	
H16	−0.368187	0.563552	0.204091	0.058*	
C17	0.1048 (19)	0.6151 (5)	0.3680 (6)	0.042 (2)	
H17	0.200289	0.589113	0.421904	0.050*	
C18	0.1843 (19)	0.7075 (5)	0.3446 (5)	0.0389 (18)	
H18	0.327922	0.743333	0.382699	0.047*	
O1W	0.7645 (12)	0.5986 (3)	0.9268 (3)	0.0503 (15)	
H1WA	0.624209	0.595327	0.962023	0.075*	
H1WB	0.768245	0.545186	0.889768	0.075*	
O2W	0.3065 (12)	0.4183 (3)	0.5379 (3)	0.0478 (14)	
H2WA	0.133161	0.418283	0.504471	0.072*	
H2WB	0.327355	0.474826	0.557396	0.072*	

### (L1) N2,N3-Bis(pyridin-4-ylmethyl)pyrazine-2,3-dicarboxamide Atomic displacement parameters (Å^2^)

**Table:**

	*U*^11^	*U*^22^	*U*^33^	*U*^12^	*U*^13^	*U*^23^
O1	0.051 (3)	0.039 (3)	0.021 (3)	0.009 (2)	0.019 (3)	0.000 (2)
O2	0.052 (3)	0.040 (2)	0.015 (3)	−0.005 (2)	0.015 (2)	−0.0003 (19)
N1	0.055 (4)	0.024 (3)	0.018 (3)	0.001 (3)	0.007 (3)	−0.002 (2)
N2	0.050 (4)	0.030 (3)	0.019 (3)	0.006 (3)	0.004 (3)	0.003 (2)
N3	0.041 (4)	0.025 (3)	0.012 (3)	0.004 (3)	0.009 (3)	−0.001 (2)
N4	0.047 (4)	0.032 (3)	0.041 (4)	0.001 (3)	0.001 (4)	0.007 (3)
N5	0.038 (4)	0.034 (3)	0.023 (3)	−0.010 (3)	0.006 (3)	−0.006 (2)
N6	0.054 (4)	0.033 (3)	0.044 (4)	−0.005 (3)	0.007 (4)	−0.003 (3)
C1	0.035 (5)	0.029 (3)	0.018 (4)	−0.002 (3)	0.009 (3)	0.001 (3)
C2	0.034 (4)	0.025 (3)	0.020 (4)	0.000 (3)	0.008 (3)	0.000 (3)
C3	0.061 (6)	0.027 (4)	0.040 (5)	0.011 (4)	0.003 (4)	0.003 (3)
C4	0.060 (5)	0.025 (4)	0.023 (4)	0.007 (4)	0.009 (4)	−0.003 (3)
C5	0.037 (4)	0.031 (4)	0.017 (4)	−0.001 (3)	0.006 (3)	0.008 (3)
C6	0.036 (4)	0.031 (4)	0.020 (4)	0.006 (3)	0.001 (3)	0.000 (3)
C7	0.032 (5)	0.030 (4)	0.027 (4)	0.013 (3)	−0.007 (4)	−0.002 (3)
C8	0.056 (5)	0.034 (4)	0.022 (5)	0.001 (4)	0.001 (4)	−0.001 (3)
C9	0.066 (6)	0.031 (4)	0.040 (5)	0.005 (4)	0.001 (5)	−0.005 (3)
C10	0.057 (6)	0.037 (4)	0.039 (5)	0.007 (4)	0.009 (4)	0.007 (4)
C11	0.053 (5)	0.035 (4)	0.024 (4)	0.002 (4)	0.006 (4)	−0.001 (3)
C12	0.047 (5)	0.027 (4)	0.015 (4)	0.007 (3)	−0.002 (4)	0.001 (3)
C13	0.047 (5)	0.029 (3)	0.025 (4)	−0.003 (3)	0.003 (4)	−0.007 (3)
C14	0.038 (5)	0.029 (3)	0.025 (5)	0.005 (3)	0.006 (4)	−0.005 (3)
C15	0.047 (5)	0.035 (4)	0.028 (4)	0.005 (4)	0.007 (4)	−0.003 (3)
C16	0.063 (6)	0.040 (4)	0.042 (5)	−0.008 (4)	0.000 (5)	−0.004 (4)
C17	0.052 (5)	0.038 (4)	0.038 (5)	0.001 (4)	0.012 (4)	0.003 (4)
C18	0.041 (5)	0.040 (4)	0.036 (5)	−0.003 (4)	0.002 (4)	−0.006 (3)
O1W	0.066 (4)	0.036 (3)	0.051 (4)	−0.006 (3)	0.013 (3)	−0.001 (2)
O2W	0.061 (4)	0.036 (3)	0.047 (3)	0.000 (2)	0.007 (3)	0.001 (2)

### (L1) N2,N3-Bis(pyridin-4-ylmethyl)pyrazine-2,3-dicarboxamide Geometric parameters (Å, º)

**Table:**

O1—C5	1.245 (7)	C6—H6B	0.9900
O2—C12	1.248 (7)	C7—C8	1.370 (9)
N1—C4	1.331 (8)	C7—C11	1.398 (9)
N1—C1	1.364 (8)	C8—C9	1.364 (10)
N2—C3	1.346 (9)	C8—H8	0.9500
N2—C2	1.351 (8)	C9—H9	0.9500
N3—C5	1.347 (8)	C10—C11	1.378 (10)
N3—C6	1.456 (8)	C10—H10	0.9500
N3—H3N	0.88 (3)	C11—H11	0.9500
N4—C10	1.330 (9)	C13—C14	1.491 (8)
N4—C9	1.354 (10)	C13—H13A	0.9900
N5—C12	1.325 (8)	C13—H13B	0.9900
N5—C13	1.458 (8)	C14—C15	1.366 (10)
N5—H5N	0.88 (3)	C14—C18	1.389 (10)
N6—C17	1.340 (10)	C15—C16	1.386 (9)
N6—C16	1.344 (10)	C15—H15	0.9500
C1—C2	1.377 (9)	C16—H16	0.9500
C1—C5	1.485 (8)	C17—C18	1.397 (10)
C2—C12	1.518 (9)	C17—H17	0.9500
C3—C4	1.375 (10)	C18—H18	0.9500
C3—H3	0.9500	O1W—H1WA	0.8569
C4—H4	0.9500	O1W—H1WB	0.9371
C6—C7	1.510 (9)	O2W—H2WA	0.8649
C6—H6A	0.9900	O2W—H2WB	0.8483
			
C4—N1—C1	115.9 (6)	C7—C8—H8	119.9
C3—N2—C2	114.8 (6)	N4—C9—C8	123.9 (7)
C5—N3—C6	122.5 (5)	N4—C9—H9	118.1
C5—N3—H3N	99 (4)	C8—C9—H9	118.1
C6—N3—H3N	139 (5)	N4—C10—C11	125.2 (7)
C10—N4—C9	115.0 (6)	N4—C10—H10	117.4
C12—N5—C13	122.6 (6)	C11—C10—H10	117.4
C12—N5—H5N	128 (4)	C10—C11—C7	118.3 (6)
C13—N5—H5N	106 (4)	C10—C11—H11	120.8
C17—N6—C16	116.6 (6)	C7—C11—H11	120.8
N1—C1—C2	120.9 (6)	O2—C12—N5	123.9 (6)
N1—C1—C5	116.8 (6)	O2—C12—C2	119.7 (6)
C2—C1—C5	122.2 (5)	N5—C12—C2	116.2 (6)
N2—C2—C1	123.1 (5)	N5—C13—C14	112.5 (6)
N2—C2—C12	111.4 (6)	N5—C13—H13A	109.1
C1—C2—C12	125.5 (6)	C14—C13—H13A	109.1
N2—C3—C4	122.5 (7)	N5—C13—H13B	109.1
N2—C3—H3	118.7	C14—C13—H13B	109.1
C4—C3—H3	118.7	H13A—C13—H13B	107.8
N1—C4—C3	122.6 (6)	C15—C14—C18	116.6 (6)
N1—C4—H4	118.7	C15—C14—C13	121.9 (6)
C3—C4—H4	118.7	C18—C14—C13	121.5 (6)
O1—C5—N3	123.0 (6)	C14—C15—C16	121.0 (7)
O1—C5—C1	120.7 (6)	C14—C15—H15	119.5
N3—C5—C1	116.3 (5)	C16—C15—H15	119.5
N3—C6—C7	112.7 (6)	N6—C16—C15	122.9 (7)
N3—C6—H6A	109.1	N6—C16—H16	118.6
C7—C6—H6A	109.1	C15—C16—H16	118.6
N3—C6—H6B	109.1	N6—C17—C18	123.0 (8)
C7—C6—H6B	109.1	N6—C17—H17	118.5
H6A—C6—H6B	107.8	C18—C17—H17	118.5
C8—C7—C11	117.3 (6)	C14—C18—C17	119.9 (7)
C8—C7—C6	121.6 (6)	C14—C18—H18	120.1
C11—C7—C6	121.1 (6)	C17—C18—H18	120.1
C9—C8—C7	120.3 (7)	H1WA—O1W—H1WB	113.1
C9—C8—H8	119.9	H2WA—O2W—H2WB	105.0
			
C4—N1—C1—C2	0.2 (9)	C7—C8—C9—N4	2.1 (12)
C4—N1—C1—C5	178.1 (6)	C9—N4—C10—C11	3.4 (12)
C3—N2—C2—C1	0.9 (10)	N4—C10—C11—C7	−1.4 (12)
C3—N2—C2—C12	179.9 (6)	C8—C7—C11—C10	−0.5 (11)
N1—C1—C2—N2	0.3 (10)	C6—C7—C11—C10	−178.9 (6)
C5—C1—C2—N2	−177.6 (6)	C13—N5—C12—O2	−4.5 (10)
N1—C1—C2—C12	−178.5 (6)	C13—N5—C12—C2	171.7 (6)
C5—C1—C2—C12	3.6 (10)	N2—C2—C12—O2	78.2 (8)
C2—N2—C3—C4	−2.5 (11)	C1—C2—C12—O2	−102.8 (8)
C1—N1—C4—C3	−1.8 (10)	N2—C2—C12—N5	−98.1 (7)
N2—C3—C4—N1	3.2 (12)	C1—C2—C12—N5	80.8 (9)
C6—N3—C5—O1	2.1 (10)	C12—N5—C13—C14	148.9 (6)
C6—N3—C5—C1	−175.4 (5)	N5—C13—C14—C15	141.5 (7)
N1—C1—C5—O1	−161.3 (6)	N5—C13—C14—C18	−40.3 (9)
C2—C1—C5—O1	16.6 (9)	C18—C14—C15—C16	1.4 (10)
N1—C1—C5—N3	16.2 (8)	C13—C14—C15—C16	179.6 (6)
C2—C1—C5—N3	−165.8 (6)	C17—N6—C16—C15	−0.6 (11)
C5—N3—C6—C7	−79.3 (7)	C14—C15—C16—N6	−0.9 (12)
N3—C6—C7—C8	94.4 (7)	C16—N6—C17—C18	1.6 (11)
N3—C6—C7—C11	−87.3 (8)	C15—C14—C18—C17	−0.4 (10)
C11—C7—C8—C9	0.1 (11)	C13—C14—C18—C17	−178.7 (6)
C6—C7—C8—C9	178.5 (7)	N6—C17—C18—C14	−1.2 (11)
C10—N4—C9—C8	−3.8 (11)		

### (L1) N2,N3-Bis(pyridin-4-ylmethyl)pyrazine-2,3-dicarboxamide Hydrogen-bond geometry (Å, º)

**Table:**

*D*—H···*A*	*D*—H	H···*A*	*D*···*A*	*D*—H···*A*
N3—H3*N*···N1	0.88 (3)	2.08 (5)	2.718 (7)	129 (5)
N5—H5*N*···O1^i^	0.88 (3)	2.05 (4)	2.858 (7)	151 (6)
O1*W*—H1*WA*···O2*W*^ii^	0.86	1.91	2.762 (7)	177
O1*W*—H1*WB*···N6^iii^	0.94	1.97	2.886 (7)	164
O2*W*—H2*WA*···O1*W*^iv^	0.86	1.91	2.765 (8)	172
O2*W*—H2*WB*···N4	0.85	2.06	2.888 (7)	164
C3—H3···O1*W*^v^	0.95	2.38	3.273 (8)	156
C4—H4···O2*W*^vi^	0.95	2.58	3.253 (8)	128
C6—H6*A*···N2^vii^	0.99	2.57	3.515 (8)	159
C16—H16···N4^iv^	0.95	2.60	3.451 (9)	149

Symmetry codes: (i) *x*−1, *y*, *z*; (ii) *x*, −*y*+1, *z*+1/2; (iii) *x*+1, −*y*+1, *z*+1/2; (iv) *x*−1, −*y*+1, *z*−1/2; (v) *x*−1, −*y*+2, *z*−1/2; (vi) *x*, *y*+1, *z*; (vii) *x*+1, −*y*+2, *z*+1/2.

### (I) Poly[[[µ4-N2,N3-bis(pyridin-4-ylmethyl)pyrazine-2,3-dicarboxamide]silver(I)] nitrate] Crystal data

**Table:**

[Ag(C_18_H_16_N_6_O_2_)]NO_3_	*D*_x_ = 1.795 Mg m^−^^3^
*M**_r_* = 518.25	Mo *K*α radiation, λ = 0.71073 Å
Orthorhombic, *F**d**d**d*	Cell parameters from 6115 reflections
*a* = 14.9776 (16) Å	θ = 1.9–25.9°
*b* = 17.3228 (12) Å	µ = 1.10 mm^−^^1^
*c* = 29.570 (4) Å	*T* = 153 K
*V* = 7672.1 (14) Å^3^	Block, colourless
*Z* = 16	0.40 × 0.30 × 0.20 mm
*F*(000) = 4160	

### (I) Poly[[[µ4-N2,N3-bis(pyridin-4-ylmethyl)pyrazine-2,3-dicarboxamide]silver(I)] nitrate] Data collection

**Table:**

Stoe IPDS 1 diffractometer	1721 independent reflections
Radiation source: fine-focus sealed tube	1038 reflections with *I* > 2σ(*I*)
Plane graphite monochromator	*R*_int_ = 0.096
φ rotation scans	θ_max_ = 25.3°, θ_min_ = 2.7°
Absorption correction: multi-scan (MULABS; Spek, 2009)	*h* = −17→17
*T*_min_ = 0.985, *T*_max_ = 1.000	*k* = −20→20
13548 measured reflections	*l* = −35→35

### (I) Poly[[[µ4-N2,N3-bis(pyridin-4-ylmethyl)pyrazine-2,3-dicarboxamide]silver(I)] nitrate] Refinement

**Table:**

Refinement on *F*^2^	Primary atom site location: structure-invariant direct methods
Least-squares matrix: full	Secondary atom site location: difference Fourier map
*R*[*F*^2^ > 2σ(*F*^2^)] = 0.045	Hydrogen site location: inferred from neighbouring sites
*wR*(*F*^2^) = 0.107	H-atom parameters constrained
*S* = 0.90	*w* = 1/[σ^2^(*F*_o_^2^) + (0.0575*P*)^2^] where *P* = (*F*_o_^2^ + 2*F*_c_^2^)/3
1721 reflections	(Δ/σ)_max_ = 0.003
141 parameters	Δρ_max_ = 0.68 e Å^−^^3^
0 restraints	Δρ_min_ = −0.65 e Å^−^^3^

### (I) Poly[[[µ4-N2,N3-bis(pyridin-4-ylmethyl)pyrazine-2,3-dicarboxamide]silver(I)] nitrate] Special details

**Table:**

Geometry. Bond distances, angles etc. have been calculated using the rounded fractional coordinates. All su's are estimated from the variances of the (full) variance-covariance matrix. The cell esds are taken into account in the estimation of distances, angles and torsion angles

### (I) Poly[[[µ4-N2,N3-bis(pyridin-4-ylmethyl)pyrazine-2,3-dicarboxamide]silver(I)] nitrate] Fractional atomic coordinates and isotropic or equivalent isotropic displacement parameters (Å^2^)

**Table:**

	*x*	*y*	*z*	*U*_iso_*/*U*_eq_	Occ. (<1)
Ag1	0.87500	0.96632 (4)	0.37500	0.0464 (2)	
O1	0.9796 (3)	0.8101 (3)	0.14979 (13)	0.0483 (14)	
N1	0.9673 (3)	0.8836 (3)	0.04722 (14)	0.0447 (18)	
N3	1.0135 (3)	0.9361 (3)	0.14261 (16)	0.050 (2)	
N4	0.9359 (3)	0.9589 (3)	0.31086 (16)	0.0427 (17)	
C1	0.9207 (3)	0.8777 (4)	0.08584 (15)	0.0337 (16)	
C4	0.9204 (4)	0.8805 (4)	0.00937 (17)	0.0453 (19)	
C5	0.9745 (3)	0.8715 (4)	0.12935 (16)	0.0340 (18)	
C6	1.0689 (4)	0.9393 (4)	0.18277 (18)	0.051 (2)	
C7	1.0197 (4)	0.9446 (3)	0.22692 (19)	0.0437 (19)	
C8	1.0640 (4)	0.9276 (4)	0.26675 (19)	0.045 (2)	
C9	1.0221 (4)	0.9354 (4)	0.3075 (2)	0.046 (2)	
C10	0.8921 (3)	0.9745 (3)	0.27201 (19)	0.043 (2)	
C11	0.9302 (4)	0.9686 (4)	0.23033 (19)	0.0447 (19)	
O11	0.9918 (8)	0.0666 (8)	0.0912 (4)	0.089 (3)	0.500
O12	1.0655 (16)	0.1323 (15)	0.1063 (6)	0.089 (3)	0.250
O13	0.9326 (9)	0.0862 (7)	0.1489 (4)	0.089 (3)	0.500
O14	0.9869 (18)	0.1233 (19)	0.1570 (7)	0.089 (3)	0.250
N10A	0.9614 (15)	0.12500	0.12500	0.089 (3)	0.500
N10B	0.9960 (16)	0.12500	0.12500	0.089 (3)	0.500
H4	0.95046	0.88600	−0.01873	0.0540*	
H3N	1.00563	0.97838	0.12661	0.0600*	
H6A	1.10904	0.98449	0.18023	0.0610*	
H6B	1.10697	0.89253	0.18345	0.0610*	
H8	1.12416	0.91024	0.26574	0.0540*	
H9	1.05428	0.92399	0.33436	0.0560*	
H10	0.83154	0.99047	0.27385	0.0520*	
H11	0.89687	0.98062	0.20392	0.0530*	

### (I) Poly[[[µ4-N2,N3-bis(pyridin-4-ylmethyl)pyrazine-2,3-dicarboxamide]silver(I)] nitrate] Atomic displacement parameters (Å^2^)

**Table:**

	*U*^11^	*U*^22^	*U*^33^	*U*^12^	*U*^13^	*U*^23^
Ag1	0.0474 (4)	0.0397 (4)	0.0521 (4)	0.0000	0.0035 (4)	0.0000
O1	0.048 (2)	0.054 (3)	0.043 (2)	0.000 (2)	−0.005 (2)	0.010 (2)
N1	0.047 (3)	0.057 (4)	0.030 (2)	0.003 (3)	0.004 (2)	−0.004 (2)
N3	0.078 (4)	0.035 (4)	0.037 (3)	0.002 (3)	−0.015 (3)	−0.006 (2)
N4	0.042 (3)	0.039 (3)	0.047 (3)	0.001 (2)	−0.009 (2)	−0.006 (2)
C1	0.037 (2)	0.035 (3)	0.029 (3)	0.005 (3)	0.002 (2)	−0.010 (3)
C4	0.059 (3)	0.047 (4)	0.030 (3)	0.009 (4)	0.006 (2)	0.000 (3)
C5	0.030 (2)	0.046 (4)	0.026 (3)	0.005 (2)	0.010 (2)	−0.007 (4)
C6	0.062 (4)	0.057 (5)	0.033 (3)	−0.003 (3)	−0.008 (3)	−0.006 (3)
C7	0.051 (3)	0.036 (4)	0.044 (3)	−0.004 (3)	−0.012 (3)	−0.008 (3)
C8	0.046 (3)	0.044 (4)	0.045 (4)	0.007 (3)	−0.006 (3)	−0.004 (3)
C9	0.051 (4)	0.051 (4)	0.037 (3)	0.009 (3)	−0.008 (3)	0.000 (3)
C10	0.037 (4)	0.041 (4)	0.052 (3)	−0.004 (3)	−0.010 (3)	−0.006 (3)
C11	0.050 (3)	0.045 (4)	0.039 (3)	−0.001 (3)	−0.015 (3)	−0.008 (3)
O11	0.112 (6)	0.088 (6)	0.067 (4)	0.0000	0.0000	0.028 (4)
O12	0.112 (6)	0.088 (6)	0.067 (4)	0.0000	0.0000	0.028 (4)
O13	0.112 (6)	0.088 (6)	0.067 (4)	0.0000	0.0000	0.028 (4)
O14	0.112 (6)	0.088 (6)	0.067 (4)	0.0000	0.0000	0.028 (4)
N10A	0.112 (6)	0.088 (6)	0.067 (4)	0.0000	0.0000	0.028 (4)
N10B	0.112 (6)	0.088 (6)	0.067 (4)	0.0000	0.0000	0.028 (4)

### (I) Poly[[[µ4-N2,N3-bis(pyridin-4-ylmethyl)pyrazine-2,3-dicarboxamide]silver(I)] nitrate] Geometric parameters (Å, º)

**Table:**

Ag1—Ag1^i^	3.1638 (11)	C8—C9	1.365 (8)
Ag1—N4	2.109 (5)	C10—C11	1.362 (8)
Ag1—O1^ii^	2.814 (5)	O11—N10A	1.493 (14)
Ag1—O1^iii^	2.814 (5)	O11—N10B	1.424 (13)
Ag1—N4^iv^	2.109 (5)	O11—O12	1.65 (3)
O1—C5	1.226 (8)	O12—N10B	1.19 (3)
N1—C1	1.342 (6)	O12—N10A	1.66 (3)
N1—C4	1.323 (7)	O13—N10A	1.066 (15)
N3—C5	1.322 (8)	O13—N10B	1.36 (2)
N3—C6	1.450 (7)	O13—O14	1.06 (3)
N4—C9	1.357 (8)	O14—N10B	0.96 (2)
N4—C10	1.350 (7)	O14—N10A	1.02 (2)
C1—C5	1.522 (6)	C4—H4	0.9500
C1—C1^v^	1.372 (6)	C6—H6B	0.9900
C4—C4^v^	1.373 (9)	C6—H6A	0.9900
N3—H3N	0.8800	C8—H8	0.9500
C6—C7	1.502 (8)	C9—H9	0.9500
C7—C8	1.384 (8)	C10—H10	0.9500
C7—C11	1.407 (8)	C11—H11	0.9500
			
N4—Ag1—N4^iv^	173.0 (2)	C6—C7—C8	119.5 (5)
O1^ii^—Ag1—O1^iii^	109.48 (14)	C6—C7—C11	123.2 (5)
Ag1^i^—Ag1—N4	86.51 (14)	C7—C8—C9	120.7 (6)
O1^ii^—Ag1—N4	97.80 (15)	N4—C9—C8	122.1 (5)
O1^iii^—Ag1—N4	86.26 (15)	N4—C10—C11	123.5 (5)
Ag1^i^—Ag1—O1^ii^	125.26 (10)	C7—C11—C10	119.1 (5)
Ag1^i^—Ag1—O1^iii^	125.26 (10)	C4^v^—C4—H4	119.00
Ag1^i^—Ag1—N4^iv^	86.51 (14)	N1—C4—H4	119.00
O1^ii^—Ag1—N4^iv^	86.26 (15)	N3—C6—H6B	108.00
O1^iii^—Ag1—N4^iv^	97.80 (15)	C7—C6—H6A	108.00
Ag1^vi^—O1—C5	114.9 (3)	H6A—C6—H6B	107.00
C1—N1—C4	116.2 (5)	C7—C6—H6B	108.00
C5—N3—C6	121.9 (5)	N3—C6—H6A	108.00
Ag1—N4—C9	119.7 (4)	C7—C8—H8	120.00
Ag1—N4—C10	122.9 (3)	C9—C8—H8	120.00
C9—N4—C10	117.4 (5)	C8—C9—H9	119.00
N1—C1—C5	116.7 (4)	N4—C9—H9	119.00
N1—C1—C1^v^	121.6 (4)	N4—C10—H10	118.00
C1^v^—C1—C5	121.6 (4)	C11—C10—H10	118.00
N1—C4—C4^v^	122.1 (5)	O11—N10A—O13	98.0 (8)
C5—N3—H3N	119.00	O13—N10A—N10B	113.9 (13)
C6—N3—H3N	119.00	O11—N10A—N10B	72.3 (9)
O1—C5—N3	124.1 (5)	O11—N10B—N10A	87.5 (11)
O1—C5—C1	120.7 (6)	O11—N10B—O13	89.0 (10)
N3—C5—C1	115.2 (5)	O13—N10B—N10A	45.8 (9)
N3—C6—C7	115.7 (5)	C7—C11—H11	120.00
C8—C7—C11	117.3 (5)	C10—C11—H11	120.00
			
Ag1^i^—Ag1—N4—C9	−71.9 (5)	N1—C1—C5—O1	−106.9 (6)
Ag1^i^—Ag1—N4—C10	106.2 (4)	N1—C1—C5—N3	73.3 (7)
O1^ii^—Ag1—N4—C9	163.0 (5)	C1^v^—C1—C5—O1	68.8 (8)
O1^ii^—Ag1—N4—C10	−19.0 (5)	C1^v^—C1—C5—N3	−111.0 (7)
O1^iii^—Ag1—N4—C9	53.8 (5)	N1—C1—C1^v^—N1^v^	5.5 (11)
O1^iii^—Ag1—N4—C10	−128.1 (5)	N1—C1—C1^v^—C5^v^	−170.1 (6)
Ag1^vi^—O1—C5—N3	−89.1 (5)	C5—C1—C1^v^—N1^v^	−170.1 (6)
Ag1^vi^—O1—C5—C1	91.1 (5)	C5—C1—C1^v^—C5^v^	14.4 (10)
C4—N1—C1—C5	172.9 (6)	N1—C4—C4^v^—N1^v^	4.5 (11)
C4—N1—C1—C1^v^	−2.9 (10)	N3—C6—C7—C8	163.0 (6)
C1—N1—C4—C4^v^	−1.9 (10)	N3—C6—C7—C11	−19.0 (9)
C6—N3—C5—O1	1.7 (8)	C6—C7—C8—C9	177.0 (6)
C6—N3—C5—C1	−178.5 (4)	C11—C7—C8—C9	−1.1 (9)
C5—N3—C6—C7	−79.7 (7)	C6—C7—C11—C10	−177.5 (6)
Ag1—N4—C9—C8	178.2 (5)	C8—C7—C11—C10	0.5 (9)
C10—N4—C9—C8	0.1 (9)	C7—C8—C9—N4	0.9 (10)
Ag1—N4—C10—C11	−178.8 (5)	N4—C10—C11—C7	0.4 (9)
C9—N4—C10—C11	−0.7 (9)		

Symmetry codes: (i) *x*, −*y*+7/4, −*z*+3/4; (ii) *x*−1/4, *y*+1/4, −*z*+1/2; (iii) −*x*+2, *y*+1/4, *z*+1/4; (iv) −*x*+7/4, *y*, −*z*+3/4; (v) −*x*+7/4, −*y*+7/4, *z*; (vi) −*x*+2, *y*−1/4, *z*−1/4.

### (I) Poly[[[µ4-N2,N3-bis(pyridin-4-ylmethyl)pyrazine-2,3-dicarboxamide]silver(I)] nitrate] Hydrogen-bond geometry (Å, º)

**Table:**

*D*—H···*A*	*D*—H	H···*A*	*D*···*A*	*D*—H···*A*
N3—H3*N*···O11^vii^	0.88	1.86	2.744 (14)	178
N3—H3*N*···O13^vii^	0.88	2.26	2.875 (13)	127
C4—H4···O11^viii^	0.95	2.45	3.378 (13)	165
C4—H4···O14^ix^	0.95	2.40	3.33 (2)	168
C9—H9···O13^x^	0.95	2.50	3.224 (14)	133
C11—H11···O13^vii^	0.95	2.51	3.154 (13)	126

Symmetry codes: (vii) *x*, *y*+1, *z*; (viii) −*x*+2, −*y*+1, −*z*; (ix) −*x*+2, *y*+3/4, *z*−1/4; (x) *x*+1/4, *y*+3/4, −*z*+1/2.
